# A complicated quasicrystal approximant ∊_16_ predicted by the strong-reflections approach

**DOI:** 10.1107/S0108768109053804

**Published:** 2010-01-22

**Authors:** Mingrun Li, Junliang Sun, Peter Oleynikov, Sven Hovmöller, Xiaodong Zou, Benjamin Grushko

**Affiliations:** a Structural Chemistry, Stockholm University, SE-106 91 Stockholm, Sweden; b Institut für Festkörperforschung, Forschungszentrum Jülich, D-52425 Jülich, Germany

**Keywords:** quasicrystal approximant, strong-reflections approach, electron diffraction, inverse Fourier transformation

## Abstract

The structure of a complicated quasicrystal approximant ∊_16_ was predicted from a known and related quasicrystal approximant ∊_6_ by the strong-reflections approach. Electron-diffraction studies show that in reciprocal space, the positions of the strongest reflections and their intensity distributions are similar for both approximants. By applying the strong-reflections approach, the structure factors of ∊_16_ were deduced from those of the known ∊_6_ structure. Owing to the different space groups of the two structures, a shift of the phase origin had to be applied in order to obtain the phases of ∊_16_. An electron-density map of ∊_16_ was calculated by inverse Fourier transformation of the structure factors of the 256 strongest reflections. Similar to that of ∊_6_, the predicted structure of ∊_16_ contains eight layers in each unit cell, stacked along the *b* axis. Along the *b* axis, ∊_16_ is built by banana-shaped tiles and pentagonal tiles; this structure is confirmed by high-resolution transmission electron microscopy (HRTEM). The simulated precession electron-diffraction (PED) patterns from the structure model are in good agreement with the experimental ones. ∊_16_ with 153 unique atoms in the unit cell is the most complicated approximant structure ever solved or predicted.

## Abstract 2

The structure of the quasicrystal approximant ∊_16_ was predicted by the strong-reflections approach based on the known approximant ∊_6_.

## 1. Introduction   

The question regarding the exact atomic positions in quasicrystals has confused crystallographers for more than two decades since the discovery of the icosahedral quasicrystal in rapidly solidified Al–Mn alloys (Shechtman *et al.*, 1984 ▸). Although quasicrystals are well ordered on the atomic scale, they are not periodic. Furthermore, the presence of defects has made it very difficult to solve their structures by single-crystal X-ray diffraction. An effective way to study the structure of quasicrystals is to start from quasicrystal approximants which often coexist with quasicrystals. This is because the latter normally have similar local atomic structures as quasicrystals. A series of quasicrystal approximants are often closely related in terms of their structures. Until today, many quasicrystal approximants have been found, but only a few of these structures have been solved by X-ray crystallography. One of the examples is the family of ∊ approximants in the Al–TM (TM = transition metal) alloys (Klein *et al.*, 1996 ▸; Sun & Hiraga, 1996 ▸; Balanetskyy, Grushko, Velikanova & Urban, 2004 ▸), where the lattice parameters *a* (≃ 23.5 Å) and *b* (≃ 16.8 Å) are essentially the same, while the *c* parameters of the regular structures are ≃12.3, 32.4, 44.9 or 57.0 Å. They are designated as ∊_6_, ∊_16_, ∊_22_, ∊_28_… (Balanetskyy, Grushko, Velikanova & Urban, 2004 ▸), where the subscript is the index *l* of the strong (00*l*) reflection corresponding to the interplanar spacing of ∼ 0.2 nm. Earlier investigations of Al–TM alloys revealed that it was difficult to synthesize Al–TM samples with only a single type of ∊ phase. The crystals often consist of different transitional states that are aperiodic in one direction. Owing to the difficulty in growing large ordered single crystals and the complexity of their structures, most of the approximant structures remain unsolved. Indeed, only the ∊_6_ structure (also known as ξ′) was solved by single-crystal X-ray diffraction (Boudard *et al.*, 1996 ▸). This situation has significantly slowed down the further investigations of quasicrystal structures.

The structures of crystals too small for single-crystal X-ray diffraction can be determined by electron crystallography. Electrostatic potential maps can be obtained by combining the structure-factor phases from HRTEM images with amplitudes from HRTEM images or electron-diffraction patterns. A successful application is to the complicated quasicrystal approximant ν-AlCrFe with space group *P*6_3_/*m*, *a* = 40.687 and *c* = 12.546 Å (Zou *et al.*, 2003 ▸). A three-dimensional electrostatic potential map was calculated by combining the structure-factor phases from HRTEM images and amplitudes from selected-area electron-diffraction (SAED) patterns of 13 zone axes. 124 of the 129 unique atoms in the unit cell were found from the electrostatic potential map. However, this method requires extensive experimental work for the determination of complicated structures such as quasicrystal approximants. First, a sufficient number of high quality images which contain phase information needs to be collected. Unfortunately, the phases from HRTEM are affected by many uncertain factors, such as defocus, astigmatism, crystal thickness and orientation. Extracting the real phases therefore relies on advanced image processing that takes into account these experimental conditions (Klug, 1978–1979 ▸; Hovmöller *et al.*, 1984 ▸; Zou *et al.*, 1996 ▸). Secondly, in order to obtain enough intensity information, many electron-diffraction patterns of different zone axes must be taken. However, as with HRTEM images, high quality kinematic electron-diffraction data are very difficult to obtain, owing to the multiple scattering of electrons (Zou & Hovmöller, 2008 ▸).

Another effective way of determining approximant structures, namely the *strong-reflections approach*, was proposed by some of the present authors (Zou & Hovmöller, 2008 ▸; Christensen *et al.*, 2004 ▸). This approach is based on the fact that the strongest reflections largely determine the atomic positions in a structure. By analyzing the relationship of the structure-factor amplitudes and phases of reflections from a series of quasicrystal approximants, we found that the strong reflections that are close to each other in reciprocal space have similar structure-factor amplitudes and phases for all the approximants in the series (Zhang, Zou *et al.*, 2006 ▸). Therefore, the structure-factor amplitudes and phases of strong reflections for an unknown approximant can be estimated from those of a known related approximant. Atomic positions in the unknown approximant are then obtained directly from the three-dimensional electron-density map calculated by inverse Fourier transformation of the structure-factor amplitudes and phases of the strong reflections. It is important to find the orientation matrix that relates the known and the unknown approximants, and re-index the reflections from the known approximant to the unknown approximant.

Structural models of five compounds in three groups of approximants (see Table 1 ▸) have been successfully deduced from their known related structures by the strong-reflections approach (Christensen *et al.*, 2004 ▸; Zhang, Zou *et al.*, 2006 ▸; Zhang, He *et al.*, 2006 ▸; He *et al.*, 2007 ▸). For all these five cases listed in Table 1 ▸, the phase origins (defined by the space groups) are the same for all structures in the same group. The structure-factor phases of the known approximant are therefore the same as those of the unknown approximant. Here we present the structure of the ∊_16_ approximant in an Al–Rh alloy, deduced by the strong-reflections approach from the known structure of the ∊_6_ approximant in the same Al–Rh system. The structure determination in the present case is more complicated since the origins in space groups *Pnma* (known structure ∊_6_) and *B*2*mm* (unknown structure ∊_16_) are different. The structure-factor phase relations of the symmetry-related reflections are not consistent in the two structures. In order to utilize their structure-factor phases, a non-standard origin was first used and then shifted to the final origin for ∊_16_. The ∊_16_ approximant has the most complicated structure ever predicted/determined by electron crystallography or the strong-reflections approach. Details are presented below.

## 2. Experimental   

Sample alloys with nominal composition Al_75_Rh_25_ and Al_77_Rh_23_ were prepared according to the Al–Rh phase diagram (Grushko *et al.*, 2000 ▸). In order to obtain ∊_6_ and ∊_16_, additional annealing was applied for up to 69 h at 1323 K to Al_75_Rh_25_ and for 2 h at 1369 K to Al_77_Rh_23_. Samples for transmission electron microscopy observation were crushed and dispersed on holey carbon films on Cu grids. Selected-area electron-diffraction (SAED) and precession electron-diffraction (PED) patterns were recorded in a Jeol 2000FX electron microscope operated at 200 kV. PED patterns of ∊_6_ and ∊_16_ were taken with a precession angle of ∼ 1.1°, using the Spinning Star Precession Unit (Avilov *et al.*, 2007 ▸). The 16-bit digitized SAED and PED patterns were analysed by the program *ELD* (Zou *et al.*, 1993 ▸). HRTEM images were recorded on a Jeol 3010 electron microscope with a point resolution of 0.17 nm operated at 300 kV. The projection symmetry of the crystal was determined from the HRTEM images by crystallographic image processing using the program *CRISP* (Hovmöller, 1992 ▸). The program *eMap* (Oleynikov, 2006 ▸) was used for calculating structure factors from the structure model, calculating three-dimensional electron-density maps from crystal structure factors, determining peak positions from the electron-density maps and simulating HRTEM images and precession electron-diffraction patterns from the structure model.

## 3. Results and discussion   

### 3.1. Unit-cell and space-group determination   

**Figure 1:**
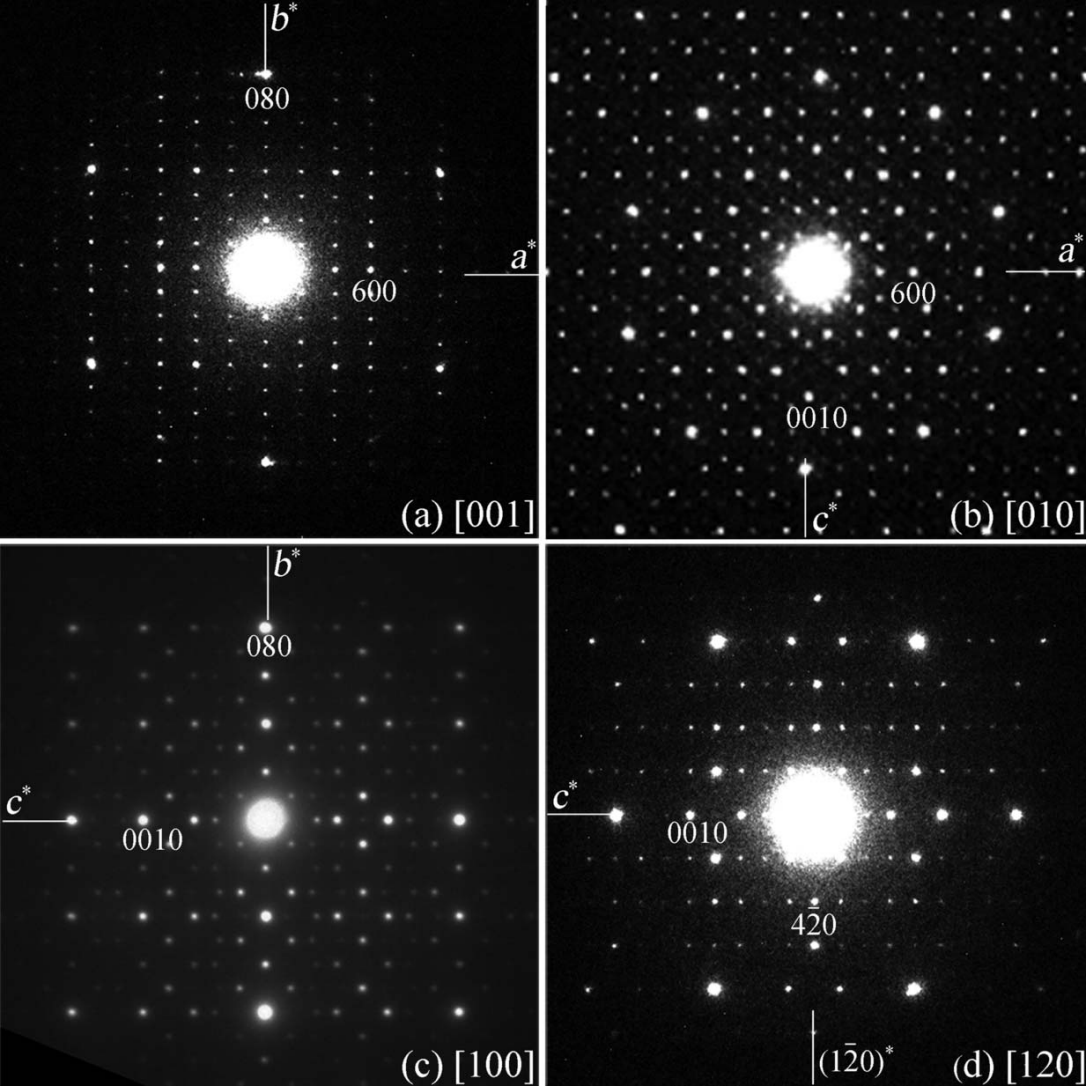
Selected-area electron-diffraction patterns of ∊_16_ along the (*a*) [001], (*b*) [010], (*c*) [100] and (*d*) [120] directions.

There are several ways to identify crystallographic features from electron microscopy. For example, by comparing the net of reflections between ZOLZ (zero-order Laue zone) and FOLZ (first-order Laue zone) that emerge at precession angles > 0.5°, the Bravais lattice and glide planes can be inferred (Morniroli *et al.*, 2007 ▸). The point group may also be deduced from convergent-beam electron-diffraction (CBED) patterns. Here we determine the space group of ∊_16_ by combining SAED and HRTEM techniques. Four electron-diffraction patterns of ∊_16_ along the [001], [010], [100] and [120] directions are shown in Fig. 1 ▸. From these, the unit cell was determined as B-centered orthorhombic with *a* = 23.5, *b* = 16.8 and *c* = 32.4 Å. ∊_16_ has almost the same *a* and *b* parameters as ∊_6_ (unit-cell parameters *a* = 23.5, *b* = 16.8, *c* = 12.3 Å), but *c* is τ^2^ times that of ∊_6_ (τ ≃ 1.618 is the golden ratio).

The reflection conditions of ∊_16_ deduced from the SAED patterns are *hkl*: *h* + *l* = 2*n*.

**Figure 2:**
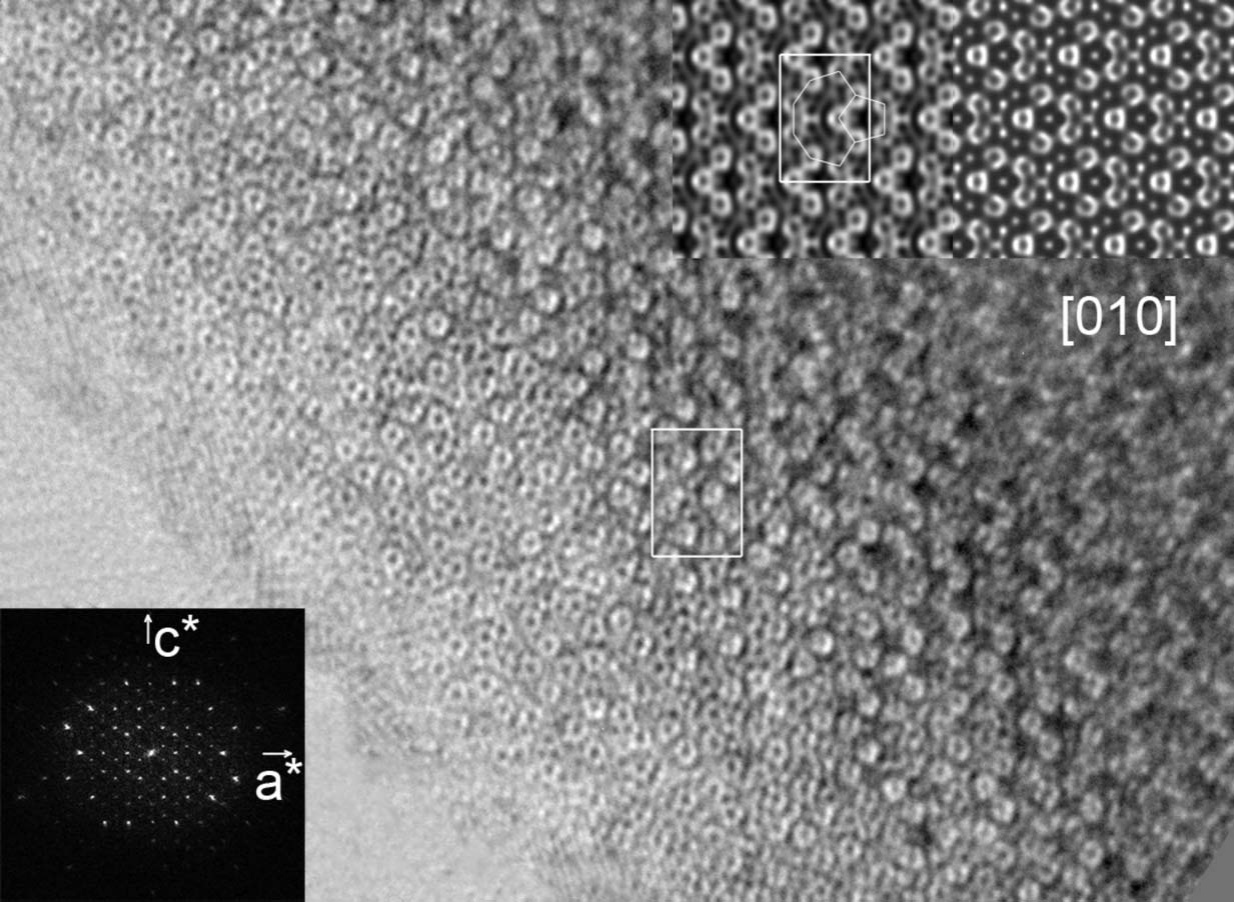
HRTEM image of ∊_16_ taken along the *b* axis. The Fourier transform of the HRTEM image, a symmetry-imposed image (left) and a simulated image (right) are inserted. There is only one mirror symmetry, perpendicular to the *c* axis. Unit cells are marked and the banana-shaped tile and the pentagonal tile are outlined in the symmetry-imposed image. The simulation was carrried out with −25 nm defocus and 6.6 nm thickness.

According to the *International Tables for Crystallography* (Hahn, 2002 ▸), four space groups – *B*222 (No. 21),* Bm*2*m* (No. 35), *B*2*mm* (No. 38) and *Bmmm* (No. 65) – fulfill these reflection conditions. These four space groups are possible for ∊_16_ and cannot be distinguished by the reflection conditions only. Fortunately, the projection symmetries along the *b* axis are different for these space groups: *cm* for *B*2*mm* and *cmm* for the others. It is possible to determine the projection symmetry of ∊_16_ by analyzing the phases extracted from the Fourier transform of the HRTEM images taken along the *b* axis. This was performed by the program *CRISP* (Hovmöller, 1992 ▸). The projection symmetry of ∊_16_ was determined as *cm* since the HRTEM image gave a much lower average phase error for *cm* (phase residual = 19.5°) than that for *cmm* (phase residual = 44.8°). This is also directly confirmed from the HRTEM image taken along [010] shown in Fig. 2 ▸. Only one mirror perpendicular to the *c* axis appears in the [010] image, and no mirror perpendicular to the *a* axis was found. Thus, the projected symmetry along [010] was determined as *cm* but not *cmm* and the only possible space group for ∊_16_ is *B*2*mm*.

### 3.2. Deducing a structure model   

**Figure 3:**
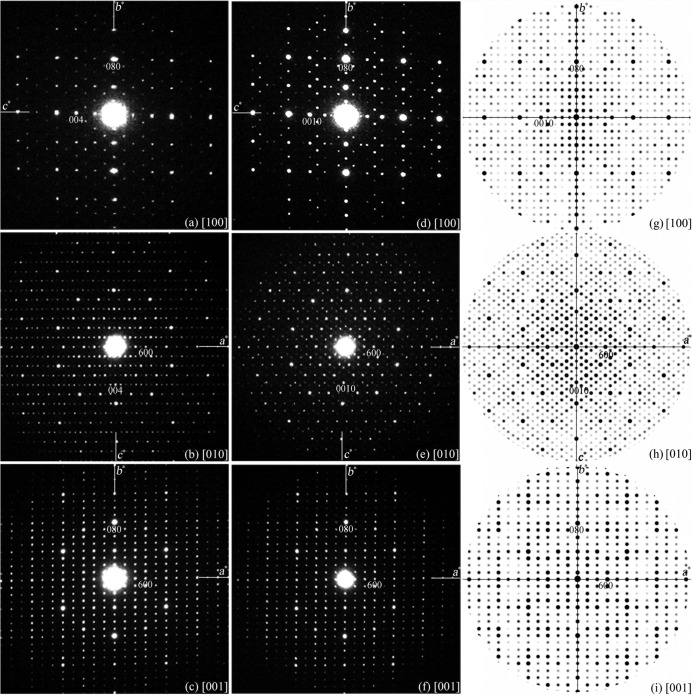
Precession electron-diffraction patterns of (*a*)–(*c*) ∊_6_ and (*d*)–(*f*) ∊_16_, and (*g*)–(*i*) the simulated precession electron-diffraction patterns from the derived structure model of ∊_16_. The kinematical simulation conditions are: voltage 200 kV, precession angle 1.1°, and crystal thicknesses (*g*) 400 Å, (*h*) 500 Å and (*i*) 400 Å. The intensity distributions in reciprocal space are similar for ∊_6_ and ∊_16_. The corresponding reflections have the same *h* and *k*, but *l*(∊_16_) ≃ τ^2^ *l*(∊_6_), such that the very strong reflection (006) in ∊_6_ corresponds to (00
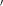
16) in ∊_16_.

Once the space group has been determined, we are ready to deduce the structure model of ∊_16_ from the structure of ∊_6_ using the strong-reflections approach. The most important condition for the strong-reflections approach is that the intensity distribution of the strongest reflections between the known and unknown structures should be similar. Thus, the first step is to identify and relate the corresponding strong reflections of ∊_16_ to those of ∊_6_ using electron diffraction. For such a purpose, precession electron-diffraction patterns were taken as shown in Fig. 3 ▸, since they are less dynamical and show higher resolution (about 0.9 Å) than those of the SAED patterns in Fig. 1 ▸. The less-dynamical diffraction intensities obtained by precession electron diffraction make the identification of the corresponding strong reflections easier. As can be seen from the experimental precession electron-diffraction patterns of ∊_6_ and ∊_16_ in Fig. 3 ▸, all the strong reflections in ∊_16_ coincide with the strong reflections in ∊_6_. Since the *a* and *b* parameters are similar for ∊_16_ and ∊_6_, and the *c* parameter of ∊_16_ (32.4 Å) is about τ^2^ times that of ∊_6_ (12.3 Å, 32.4/12.3 = 2.634), the (*hkl*) indices of the strong reflections in these two approximants are related by (*h k l*_∊16_) = (*h k* τ^2^ *l*_∊6_). Here, the golden number τ = (1 + 5^1/2^)/2 is associated with fivefold rotational symmetry of an icosahedral quasicrystal. Elser *et al.* (Elser & Henley, 1985 ▸) used the rational ratio of two successive Fibonacci numbers (1, 1, 2, 3, 5, 8, 13, 21, …, *F*_*n*_, …; *F*_*n*_ = *F*_*n* − 1_ + *F*_*n* − 2_) as an approximation to substitute for the irrational τ to obtain the crystalline approximant of an icosahedral quasicrystal. According to the Fibonacci series, (*h k* 8) in ∊_16_ is related to (*h k* 3) in ∊_6_, (*h k* 10) in ∊_16_ is related to (*h k* 4) in ∊_6_, (*h k* 26) in ∊_16_ is related to (*h k* 10) in ∊_6_, and so on.

**Figure 4:**
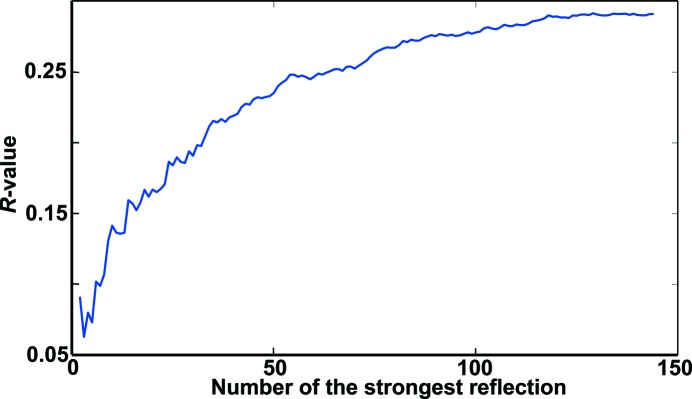
*R* values against the number of corresponding strongest reflections in ∊_6_ and ∊_16_. For the 30 strongest reflections, an *R* value of 0.19 shows good correspondence for the strong reflections in ∊_6_ and ∊_16_. As more and more moderately strong reflections are included, the *R* value increases, reaching 0.29 for the 146 strongest reflections.

To confirm the similarity of the ∊_16_ and ∊_6_ structures, the *R* value against the number of corresponding strongest reflections was plotted in Fig. 4 ▸ without any corrections (such as absorption correction, Lorenz correction and so on). The detailed reflection lists are given as supporting information.^1^ The reflections from different orientations were merged using their common reflections. It shows that the intensities of the strong corresponding reflections are very close to each other (*R* = 0.19) for the 30 strongest reflections. As more and more moderately strong reflections are included, the *R* value increases, reaching 0.29 for the 146 strongest reflections. This kind of *R* value is close to a typical internal *R* value for elctron-diffraction data obtained from different particles of the same structure. Thus, we think there is obvious similarity in the ∊_16_ and ∊_6_ structures. Note that here we only compared the experimental strong reflections along the three main zone axes, but they constitute a major part of all data. For the 256 strongest reflections (the 45 strongest of which are listed in Table 2 ▸), the amplitudes along these three main zone axes sum up to 45% of the total amplitudes in ∊_6_.

The strong reflections in ∊_16_ are then deduced from the corresponding reflections in the known ∊_6_ according to the relations described above. In order to determine the number of independent strong reflections that are needed to obtain a sufficiently good electron-density map, we first checked the procedure on the ∊_6_ structure. Generally speaking, the structure-factor amplitude sum of the strong reflections must be more than 50% of the total amplitude sum in order to generate an electron-density map that represents the structure (Zhang, He *et al.*, 2006 ▸). Here we choose the 256 strongest reflections of ∊_6_ among the total 2640 independent reflections within 1.0 Å resolution. Table 2 ▸ lists 45 of them, together with their structure-factor amplitudes and phases. The 256 reflections sum up to 57% of the total amplitude and they were expanded to 1590 reflections according to the symmetry of ∊_6_. A three-dimensional electron-density map was calculated from the structure-factor amplitudes and phases of these 1590 reflections by inverse Fourier transformation, using the program *eMap* (Oleynikov, 2006 ▸). All the atomic positions of ∊_6_ as determined by single-crystal X-ray diffraction (Boudard *et al.*, 1996 ▸) could be found in the three-dimensional density map. This indicates that the 256 strong reflections are sufficient to obtain a correct structure model of ∊_6_. Since the strong reflections in ∊_16_ coincide with the strong reflections in ∊_6_, a reasonable structure model of ∊_16_ should be obtained using the 256 strong reflections of ∊_16_ deduced from those of ∊_6_.

The structure-factor amplitudes of ∊_16_ were assigned from those of the corresponding reflections in ∊_6_, calculated from the single-crystal X-ray structure model (Table 2 ▸). We did not use the amplitudes from the experimental PED data since it was difficult to collect a complete three-dimensional PED data due to mixed phases in the same particle. A new technique, called electron-diffraction tomography, is being developed in our department (Zhang *et al.*, 2010 ▸) and may be applied in the future for collecting complete three-dimensional electron-diffraction data. Our earlier studies have shown that the amplitudes taken from the corresponding known approximants are enough to deduce the structure model.

Different from our previous studies (Christensen *et al.*, 2004 ▸; Zhang, He *et al.*, 2006 ▸; Zhang, Zou *et al.*, 2006 ▸; He *et al.*, 2007 ▸), the structure-factor phases for the strong reflections of ∊_16_ cannot be taken directly from those of ∊_6_, since the choice of origin for the space group of ∊_16_ (*B*2*mm*) is different from that of the space group for ∊_6_ (*Pnma*). Consequently, the phase relations between the symmetry-related reflections in the two space groups are different. For the space group *Pnma*, the relations are (Table 2 ▸):

(i) If *h* + *l* = 2*n* and *k* = 2*n*

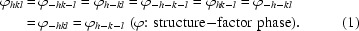

(ii) If *h* + *l* = 2*n* and *k* = 2*n* + 1:
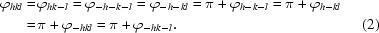

(iii) If *h* + *l* = 2*n* + 1 and *k* = 2*n*:
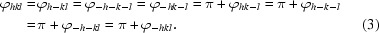

(iv) If *h* + *l* = 2*n* + 1 and *k* = 2*n +*1:
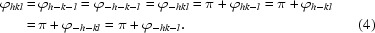

For the space group *B*2*mm*, the relations are simpler
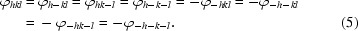



One way to overcome the problem of the different phase relationships is to first assume the space group *P*1 for ∊_16_. Starting from 256 strong reflections in ∊_6_, they are expanded into 1590 reflections using *Pnma* symmetry. Based on the strong-reflection approach, each of these strong reflections in ∊_6_ structure has one corresponding reflection in the ∊_16_ structure in *P*1 symmetry with the same phases and amplitudes but different indices [see Table 2 ▸, column Phase ∊_6_ (*Pnma*) and ∊_16_ (*P*1)]. This ∊_16_ structure in *P*1 symmetry turns out to be very close to *B*2*mm* symmetry. Note that although the phase relations between the symmetry-related reflections in the two space groups *Pnma* and *B*2*mm* are different, the phase relations for the strongest reflections in the two structures are almost the same.

**Figure 5:**
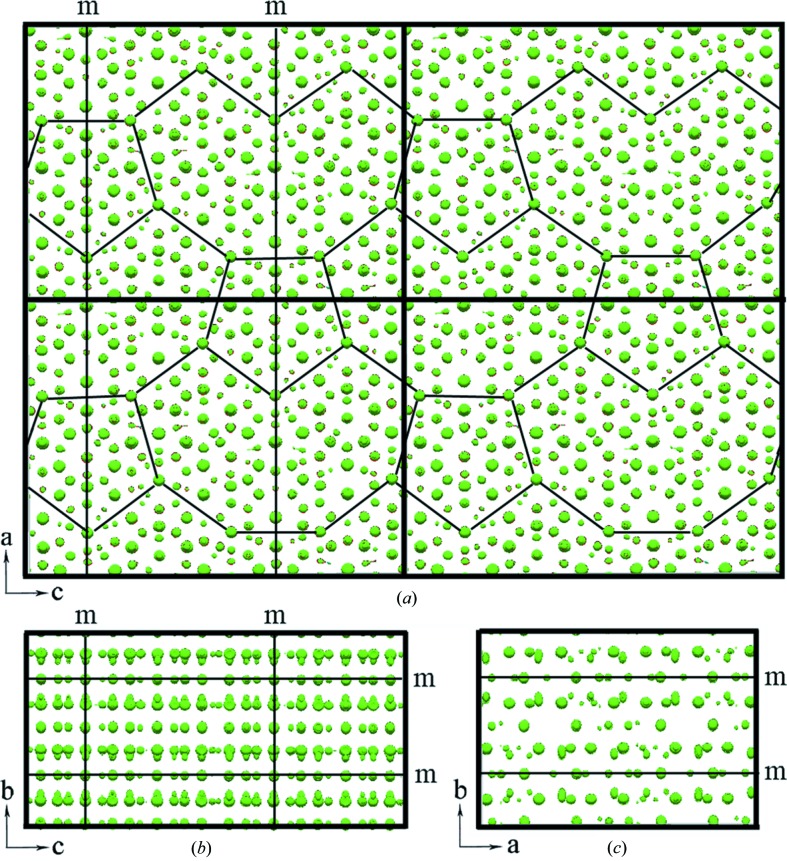
A three-dimensional electron-density map of ∊_16_ calculated from 1590 (256 independent) strong reflections using the space group *P*1. (*a*), (*b*) and (*c*) are three-dimensional density maps viewed along the *b*, *a* and *c* axes, respectively. Four unit cells are outlined in (*a*). The symmetry elements can be identified from the density map, with mirrors perpendicular to the *b* and *c* axes (marked). The new origin is set on 2*mm*. The origin shifts obtained from the electron-density maps are Δ*x* = 0, Δ*y* = 0.25 and Δ*z* = 0.15625. Banana-shaped clusters and pentagonal clusters^21^ are outlined in (*a*).

The three-dimensional electron-density map of ∊_16_ in *P*1 symmetry gives well resolved peaks that can be assigned to atomic positions, as shown in Fig. 5 ▸. From the density map viewed along the *b* axis (Fig. 5 ▸ *a*), the banana-shaped tiles and pentagonal tiles (Balanetskyy, Grushko & Velikanova, 2004 ▸) can be identified. The two types of tiles are alternating along the *a* and *c* directions and connected to each other. Similar tiles and connections are observed in the [010] HRTEM image in Fig. 2 ▸. The symmetries can be identified from the three-dimensional electron-density map as follows: *B*-centering, 2 // ***a***, *m* ⊥ ***b***, *m* ⊥ ***c***, which agrees with the space group *B*2*mm.* An origin that is compatible with the space group *B*2*mm* was found at a 2*mm* Wyckoff position, (0, 0.25, 0.15625). Thus, the origin was shifted to this position and the new structure-factor phases were calculated using the following equation
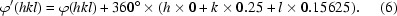



The new structure factor phases of 45 reflections from the 256 independent strong reflections are listed in Table 2 ▸, together with the symmetry-imposed phases. The average deviation of the phases from the symmetry *B*2*mm* is only 7.8°, and the largest phase error is 22.5°. The amplitudes together with the phases of the 256 reflections after imposing the symmetry *B*2*mm* were used to calculate a new three-dimensional electron-density map of ∊_16_. The three-dimensional density map is very similar to that with *P*1 symmetry, but the electron densities at symmetry-related positions become exactly identical instead of just similar to each other.

There were 150 unique peaks corresponding to the atomic positions of ∊_16_ identified from the three-dimensional density map and the atomic coordinates were determined. There were 33 of them assigned as Rh, and the remaining 117 were Al. The assignment of Rh positions was based on:

(i) the peak height (the highest peaks) and
(ii) chemical knowledge.

Since ∊_16_ and ∊_6_ are both members of the same series of icosahedral quasicrystal approximants, they are expected to have very similar local atomic structures. Atoms in similar clusters should have a similar environment, and thus atoms in ∊_16_ were assigned to form similar clusters as those in ∊_6_. In addition, three Al atoms were added to complete the structure based on the geometry and similarity to ∊_6_, see Table 3 in the supplementary material. Most of the atoms have reasonable distances to their neighbors, ranging from 2.2 to 3.1 Å. The final composition of our ∊_16_ model is Al_340_Rh_99_, which fits the synthesis stoichiometry (Al_77_Rh_23_) very well.

The precession electron-diffraction patterns simulated using the derived ∊_16_ structure model (Figs. 3 ▸ *g*–*i*) agree well with the experimental precession electron-diffraction patterns (Figs. 3 ▸ *d*–*f*). A least-squares refinement was performed with only four refined parameters (overall scale factor, extinction parameters and isotropic atomic displacement parameters for Rh and Al) using the experimental ∊_16_ intensities from PED patterns (659 independent reflections within 1.0 Å resolution) in the *SHELXL* program (Sheldrick, 2008 ▸). The refinement converged with *R*_1_ = 0.33. Thus, the structure model deduced for ∊_16_ from ∊_6_ can be taken as a good preliminary model.

### 3.3. Structure description   

**Figure 6:**
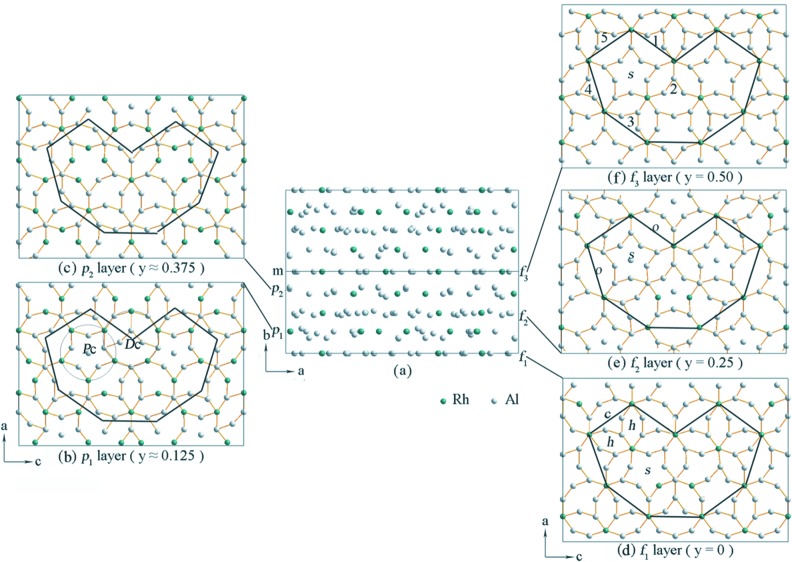
The atomic structure of ∊_16_ (*a*) viewed along the *c* axis. Eight layers, including four flat layers (*f*) and four puckered layers (*p*), are stacked along the *b* axis. Five independent layers are identified; at *y* = 0 (*f*_1_), ∼ 0.125 (*p*_1_), 0.25 (*f*_2_), ∼ 0.375 (*p*_2_) and 0.5 (*f*_3_). The structure can be described as 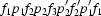, where 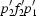 are obtained from the layers *p*_1_ *f*_2_ *p*_2_ by a mirror symmetry at *y* = 0.5. (*b*)–(*f*) The five independent layers viewed along the *b* axis. Three basic tilings: squashed hexagon (*h*), pentagonal star (*s*) and crown (*c*) are indicated in (*d*). A decagonal column (*D*c) and a pentagonal column (*P*c) are marked in (*b*). A squashed octagon (*o*) is marked in (*e*). A pentagonal star (*s*) surrounded by five hexagonal columns is marked in (*f*).

The final structure of ∊_16_ viewed along the *c* axis is shown in Fig. 6 ▸(*a*). There are eight layers that are perpendicular to the *b* axis in each unit cell, including four flat (*f*) and four puckered (*p*) layers. Five of the layers, at *y* = 0 (*f*_1_), ∼ 0.125 (*p*_1_), ∼ 0.25 (*f*_2_), ∼ 0.375 (*p*_2_) and 0.5 (*f*_3_), are independent (Figs. 6 ▸ *b*–*f*). The other three can be generated by a mirror located at *y* = 0.5. The atoms within the *f*_2_ layer deviate slightly from *y* = 0.25. Since this deviation is very small (< 0.019), *f*_2_ is still considered as a flat layer. Therefore, the structure can be described as 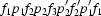, where 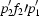 are related to the layers *p*_1_ *f*_2_ *p*_2_ by a mirror operation at *y* = 0.5. Each layer contains three common basic tilings: a squashed hexagon (*h*), a pentagonal star (*s*) and a crown (*c*) (marked in Fig. 6 ▸ *d*). Two *h* tilings together with one *c* tiling form a decagonal ring. Each *s* tiling is surrounded by five inter-connected decagonal rings (marked in Fig. 6 ▸ *f*). An additional eight-ring tiling (*o*) is also found in the layer *f*_2_ (marked in Fig. 6 ▸ *e*).

**Figure 7:**
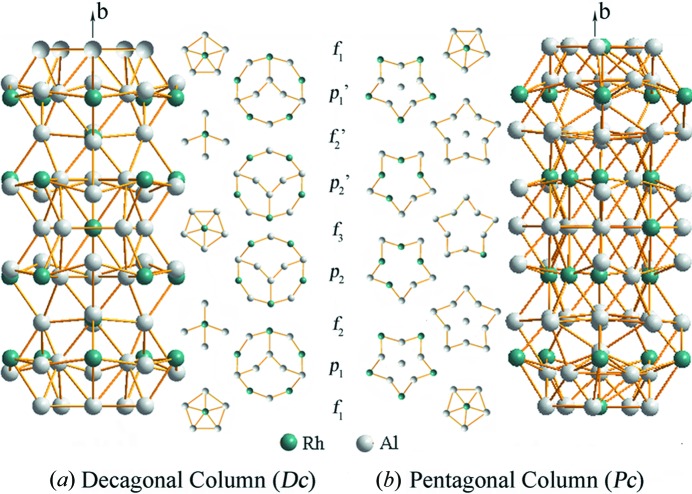
Two types of columns related to decagonal quasicrystals. (*a*) Side view of the decagonal column. Each puckered layer contributes a decagonal ring to the decagonal column. Between the decagonal rings are small pentagons (in *f*_1_ and *f*_3_) and irregular subunits (in *f*_2_) that are alternating along the *b* axis. (*b*) Side view of the pentagonal column. The pentagonal column is constructed by pentagonal stars or pentagons that are stacked along the *b* axis. The centers of the decagonal columns are always occupied by the heavy Rh atoms.

The structure of ∊_16_ can be described using two types of columns along the *b* axis: a decagonal column (*D*c) and a pentagonal column (*P*c) (marked in Fig. 6 ▸ *b*). The construction of the decagonal and pentagonal columns is given in Fig. 7 ▸. Each puckered layer contributes a decagonal ring to the decagonal column. Between the decagonal rings are small pentagons (in *f*_1_ and *f*_3_) and irregular tilings (in *f*_2_) that are alternating along the *b* axis (Fig. 7 ▸ *a*). The pentagonal column is constructed by pentagonal stars or pentagons that are stacked along the *b* axis (Fig. 7 ▸ *b*). The centers of the decagonal columns are always occupied by the heavy Rh atoms. The decagonal columns are connected to each other through edge-sharing (*via* two common atoms). Five decagonal columns form a pentagonal tile with a pentagonal column inside (Fig. 6 ▸ *b*). Nine decagonal columns form a banana-shaped tile with two pairs of intersecting pentagonal columns inside. The banana-shaped and pentagonal tiles are seen in the HRTEM images (see Fig. 2 ▸). Similar decagonal and pentagonal columns have also been observed in the decagonal quasicrystal d-Al–Pd (Li *et al.*, 1996 ▸).

**Figure 8:**
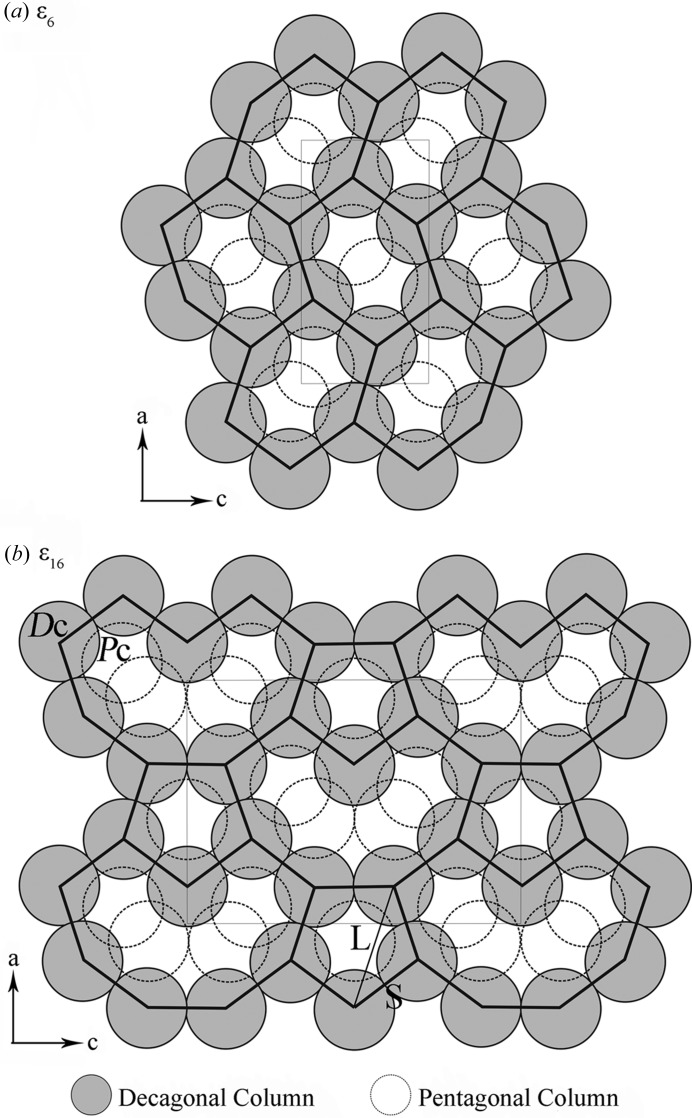
The construction of ∊_6_ and ∊_16_ from the decagonal columns (*D*c) and pentagonal columns (*P*c) running along the *b* axis. The structure of ∊_6_ is constructed by only one type of hexagonal tiles (outlined) built from six decagonal columns with two intersecting pentagonal columns inside. The structure of ∊_16_ is constructed by two types of tiles alternating along the *a* and *c* axes (outlined): a banana-shaped tile and a pentagonal tile.

A comparison of the structure features of ∊_6_ and ∊_16_ is given in Fig. 8 ▸. Both structures can be described by the decagonal and pentagonal columns. All decagonal columns in ∊_6_ and ∊_16_ are connected by edge-sharing. The structure of ∊_6_ is constructed by only one type of hexagonal tile built from six decagonal columns with two intersecting pentagonal columns inside. The structure of ∊_16_ is constructed by two types of tiles: a banana-shaped tile and a pentagonal tile. The diameter of decagonal and pentagonal columns is about 7.6 Å in both structures. This is also the edge length (marked by thick lines in Fig. 8 ▸) of the different tiles. We designate this edge length as ‘S’, as the short distance in a Fibonacci series as found in quasicrystal structures. The diagonal distance of the pentagonal tile is τ times longer than S, and thus defined as ‘L’. As shown in Fig. 8 ▸, the *c* parameters can be given by S and L as follows: ∊_6_: *c* = L = τS ≃ 12.3 Å; ∊_16_: *c* = S + L + L ≃ 32.2 Å. Consequently the *c* parameters for the other approximants in the series are: ∊_22_: *c* = S + L + L + L ≃ 44.5 Å; ∊_28_: *c* = S + L + L + L + L ≃ 56.8 Å. They are related by 1: (1 + τ): (2 + τ): (3 + τ).

We expect that it will be possible to obtain the structures of the complete ∊ series and describe them using the stacks of decagonal and pentagonal columns. Furthermore, a detailed structure model of the decagonal quasicrystal (d-Al–TM) can possibly be deduced from these common features.

## 4. Conclusion   

A series of approximants of quasicrystals are expected to be built from similar atomic clusters, resulting in similar intensity distributions of reflections in reciprocal space. The similarity of the intensity distributions leads to similar structure-factor amplitudes and phases of the related reflections, especially for the strongest reflections. Based on this knowledge, the structure-factor amplitudes and phases of an unknown structure can be deduced from those of a related known structure. A structure model can be deduced from the three-dimensional electron-density map obtained by inverse Fourier transformation of the structure factors. Such a case was demonstrated here successfully on the complex and unknown structure of ∊_16_ based on the related known structure of ∊_6_. The derived structure model agrees well with the experimental precession electron-diffraction patterns and high-resolution transmission electron microscopy images. The structure of ∊_16_ with 153 unique atoms within the unit cell is the most complex quasicrystal approximant predicted or solved so far. The strong-reflections approach has once again proven to be an effective method for predicting and solving unknown quasicrystal approximants.

## Supplementary Material

Tables of coordinates and experimental intensities of epsilon_6 and 16. DOI: 10.1107/S0108768109053804/dr5024sup1.pdf

## Figures and Tables

**Table 1:** List of quasicrystal approximants solved by the strong-reflections approach

Known structure							Deduced structure						
		Lattice parameters ()							Lattice parameters ()				
Phase	Space group	*a* ()	*b* ()	*c* ()	()		Phase	Space group	*a* ()	*b* ()	*c* ()	()	Ref.
*m*-Al_13_Co_4_	*C*2/*m*	15.2	8.1	12.4	108		^2^-Al_13_Co_4_	*P*2/*m*	39.9	8.1	32.2	108	Christensen *et al.* (2004 ▸)
_3_-ZnMgRE	*P*6_3_/*mmc*	14.6		8.6		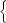	_5_-ZnMgRE	*P*6_3_/*mmc*	23.5		8.6		Zhang, Zou *et al.* (2006 ▸)
							_7_-ZnMgRE	*P*6_3_/*mmc*	33.6		8.9		
-Al_4_Mn	*P*6_3_/*m*	28.38		12.39		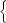	()-AlCrSi	*P*6_3_/*mmc*	32.3		12.4		Zhang, He *et al.* (2006 ▸)
							-AlCrSi	*P*6_3_/*mmc*	20.1		12.4		He *et al.* (2007 ▸)
_6_	*Pnma*	23.5	16.8	12.3			_16_	*B*2*mm*	23.5	16.8	32.4		This work

**Table 2:** List of structure-factor amplitudes and phases of 45 strongest independent reflections of _6_ (with *Pnma*) and _16_ (with *P*1 and *B*2*mm*) After the origin has been shifted from that of _6_ to (0, 0.25, 0.15625), the phases of symmetry-related reflections [Phase _16_ (*P*1)] are close to those required by the space group *B*2*mm*, with an average phase error of 7.8 and a maximum phase error of 22.5 compared with phases after imposing the *B*2*mm* symmetry.

			Amplitude	Phase											Phase _16_ (*B*2*mm*)
_6_			_6_ and _16_	_6_ (*Pnma*) and _16_				_16_			Origin shifted (0, 0.025, 0.15625)				Symmetry-imposed
*h*	*k*	*l*		*hkl*	*hk* *l*	*h* *k* *l*	*h* *kl*	*h*	*k*	*l*	*hkl*	*h* *kl*	*hk* *l*	*h* *k* *l*	*hkl*
0	8	0	2815	0	0	0	0	0	8	0	0	0	0	0	0
0	0	10	2338	0	0	0	0	0	0	26	23	23	338	338	0
8	4	3	2279	0	180	180	0	8	4	8	90	90	90	90	90
18	0	3	2262	180	0	0	180	18	0	8	270	270	270	270	270
10	4	0	2259	180	180	180	180	10	4	0	180	180	180	180	180
11	0	8	2211	180	0	0	180	11	0	21	281	281	259	259	270
3	4	5	2205	180	180	180	180	3	4	13	191	191	169	169	180
7	0	5	2036	0	0	0	0	7	0	13	11	11	349	349	0
11	0	2	2021	0	180	180	0	11	0	5	281	281	259	259	270
0	0	6	2018	180	180	180	180	0	0	16	0	0	0	0	0
0	16	0	1567	0	0	0	0	0	16	0	0	0	0	0	0
26	4	0	1471	0	0	0	0	26	4	0	0	0	0	0	0
8	4	13	1471	0	180	180	0	8	4	34	113	113	68	68	90
0	8	10	1457	0	0	0	0	0	8	26	23	23	338	338	0
18	8	3	1454	180	0	0	180	18	8	8	270	270	270	270	270
21	4	8	1433	0	180	180	0	21	4	21	101	101	79	79	90
11	8	8	1406	180	0	0	180	11	8	21	281	281	259	259	270
8	12	3	1288	0	180	180	0	8	12	8	90	90	90	90	90
13	7	5	1275	0	0	180	180	13	7	13	281	281	259	259	270
19	4	5	1269	0	0	0	0	19	4	13	11	11	349	349	0
16	7	0	1269	0	0	180	180	16	7	0	270	270	270	270	270
10	4	10	1256	180	180	180	180	10	4	26	203	203	158	158	180
5	7	8	1241	0	180	0	180	5	7	21	11	11	349	349	0
0	4	0	1234	180	180	180	180	0	4	0	180	180	180	180	180
15	4	8	1216	0	180	180	0	15	4	21	101	101	79	79	90
10	12	0	1194	180	180	180	180	10	12	0	180	180	180	180	180
0	8	6	1181	180	180	180	180	0	8	16	0	0	0	0	0
21	4	2	1178	180	0	0	180	21	4	5	101	101	79	79	90
3	4	11	1169	0	0	0	0	3	4	29	191	191	169	169	180
5	7	2	1163	180	0	180	0	5	7	5	11	11	349	349	0
3	12	5	1162	180	180	180	180	3	12	13	191	191	169	169	180
11	8	2	1151	0	180	180	0	11	8	5	281	281	259	259	270
7	8	5	1126	0	0	0	0	7	8	13	11	11	349	349	0
18	0	7	1057	0	180	180	0	18	0	18	293	293	248	248	270
2	7	3	1038	0	180	0	180	2	7	8	0	0	0	0	0
0	14	0	1035	180	180	180	180	0	14	0	0	0	0	0	0
8	11	3	1010	180	0	180	0	8	11	8	180	180	180	180	180
7	0	11	998	180	180	180	180	7	0	29	11	11	349	349	0
10	0	0	973	0	0	0	0	10	0	0	0	0	0	0	0
15	4	2	970	180	0	0	180	15	4	5	101	101	79	79	90
10	11	0	961	180	180	0	0	10	11	0	90	90	90	90	90
3	11	5	959	180	180	0	0	3	11	13	101	101	79	79	90
22	0	0	955	180	180	180	180	22	0	0	180	180	180	180	180
8	4	7	952	180	0	0	180	8	4	18	113	113	68	68	90
19	12	5	903	0	0	0	0	19	12	13	11	11	349	349	0

## Footnotes

1 Supplementary data for this paper are available from the IUCr electronic archives (Reference: DR5024). Services for accessing these data are described at the back of the journal.
