# Supplementary material for: A complicated quasicrystal approximant ∊16 predicted by the strong-reflections approach
Source: Acta Crystallogr B. 2010 Jan 22;66(Pt 1):17–26. doi: 10.1107/S0108768109053804 (PMC2811402; doi:10.1107/S0108768109053804)
Supplement: Supplementary file 1 [file b-66-00017-sup1.pdf]

Table 3 Atomic coordinates of ε_{16} in Al-Rh systems.

Atom	x	y	z	Atom	x	y	z	Atom	x	y	z
Rh1	0.156	0.000	0.000	Al19	0.26	0.000	0.382	Al70	0.067	0.152	0.19
Rh2	0.07	0.000	0.191	Al20	0.07	0.000	0.427	Al71*	0.706	0.134	0.5
Rh3	0.344	0.000	0.193	Al21	0.454	0.000	0.427	Al72	0.087	0.134	0.383
Rh4	0.156	0.000	0.382	Al22	0.036	0.000	0.5	Al73	0.1	0.094	0.12
Rh5	0.343	0.000	0.427	Al23	0.38	0.000	0.5	Al74	0.1	0.094	0.265
Rh6	0.158	0.246	0.000	Al24	0.046	0.232	0.000	Al75	0.1	0.094	0.5
Rh7	0.342	0.248	0.19	Al25	0.036	0.244	0.12	Al76	0.175	0.094	0.19
Rh8	0.158	0.246	0.385	Al26	0.036	0.244	0.262	Al77	0.175	0.094	0.427
Rh9	0.342	0.248	0.43	Al27	0.036	0.244	0.5	Al78	0.206	0.134	0.354
Rh10	0.156	0.5	0.000	Al28	0.07	0.26	0.191	Al79	0.295	0.114	0.075
Rh11	0.156	0.5	0.381	Al29	0.07	0.26	0.43	Al80	0.286	0.134	0.16
Rh12	0.346	0.5	0.19	Al30	0.12	0.234	0.074	Al81	0.295	0.134	0.31
Rh13	0.43	0.5	0.000	Al31	0.12	0.234	0.312	Al82	0.295	0.134	0.456
Rh14	0.43	0.5	0.381	Al32	0.158	0.246	0.146	Al83	0.32	0.134	0.237
Rh15	0.02	0.134	0.073	Al33	0.158	0.246	0.238	Al84	0.4	0.134	0.16
Rh16	0.02	0.134	0.309	Al34	0.238	0.234	0.19	Al85	0.4	0.134	0.458
Rh17	0.018	0.134	0.456	Al35	0.238	0.234	0.428	Al86	0.483	0.094	0.118
Rh18	0.206	0.134	0.119	Al36	0.26	0.258	0.000	Al87	0.483	0.094	0.266
Rh19	0.206	0.134	0.266	Al37	0.27	0.244	0.5	Al88	0.485	0.09	0.354
Rh20	0.206	0.134	0.5	Al38	0.34	0.248	0.046	Al89	0.483	0.094	0.5
Rh21	0.324	0.134	0.000	Al39	0.342	0.248	0.338	Al90	0.016	0.404	0.074
Rh22	0.32	0.134	0.38	Al40	0.378	0.26	0.118	Al91	0.016	0.394	0.308
Rh23	0.4	0.134	0.072	Al41	0.378	0.26	0.5	Al92	0.016	0.404	0.457
Rh24	0.4	0.134	0.309	Al42	0.43	0.234	0.000	Al93	0.1	0.364	0.354
Rh25	0.1	0.364	0.12	Al43	0.43	0.234	0.238	Al94	0.176	0.364	0.048
Rh26	0.1	0.364	0.264	Al44	0.43	0.234	0.385	Al95	0.176	0.364	0.43
Rh27	0.1	0.364	0.5	Al45	0.47	0.246	0.073	Al96	0.205	0.384	0.119
Rh28	0.176	0.364	0.192	Al46	0.46	0.246	0.309	Al97	0.208	0.384	0.264
Rh29	0.29	0.364	0.074	Al47	0.46	0.246	0.454	Al98	0.208	0.374	0.356
Rh30	0.292	0.364	0.31	Al48	0.048	0.5	0.000	Al99	0.208	0.384	0.5
Rh31	0.48	0.365	0.118	Al49	0.038	0.5	0.12	Al101	0.29	0.364	0.16
Rh32	0.48	0.365	0.264	Al50	0.048	0.5	0.236	Al102	0.292	0.364	0.456
Rh33	0.48	0.365	0.5	Al51	0.048	0.5	0.381	Al103	0.325	0.404	0.000
Al1	0.26	0.000	0.000	Al52	0.038	0.5	0.5	Al104	0.324	0.404	0.236
Al2	0.07	0.000	0.046	Al53	0.12	0.5	0.074	Al105	0.325	0.404	0.385
Al3	0.342	0.000	0.046	Al54	0.12	0.5	0.31	Al106	0.398	0.402	0.074
Al4	0.185	0.000	0.075	Al55	0.12	0.5	0.456	Al107	0.398	0.402	0.31
Al5	0.46	0.000	0.073	Al56	0.156	0.5	0.146	Al108	0.398	0.406	0.456
Al6	0.038	0.000	0.118	Al57	0.156	0.5	0.238	Al109	0.41	0.362	0.191
Al7	0.378	0.000	0.118	Al58	0.24	0.5	0.044	Al110	0.43	0.346	0.000
Al8	0.154	0.000	0.146	Al59	0.24	0.5	0.19	Al111	0.48	0.365	0.354
Al9	0.26	0.000	0.146	Al60	0.238	0.5	0.426	Al112	0.186	0.000	0.456
Al10	0.454	0.000	0.193	Al61	0.313	0.5	0.118	Al113	0.27	0.000	0.5
Al11	0.156	0.000	0.238	Al62	0.318	0.5	0.266	Al114	0.222	0.24	0.306
Al12	0.258	0.000	0.238	Al63	0.318	0.5	0.5	Al115	0.24	0.5	0.34
Al13	0.036	0.000	0.266	Al64	0.344	0.5	0.048	Al116	0.34	0.5	0.34
Al14	0.378	0.000	0.266	Al65	0.345	0.5	0.426	Al117	0.46	0.5	0.456
Al15	0.185	0.000	0.31	Al66	0.43	0.5	0.146	Al118	0.134	0.122	0.05
Al16	0.46	0.000	0.3	Al67	0.43	0.5	0.238	Al119*	0.6	0.364	0.5
Al17	0.07	0.000	0.335	Al68	0.46	0.5	0.074	Al120*	0.309	0.246	0.727
Al18	0.343	0.000	0.338	Al69	0.46	0.5	0.31				

* These three Al positions were added based on the geometry and similarity to ε_6.

Table S1 Experimental intensities of ε_6 and ε_{16}

H	K	L	$I_{\varepsilon 6}$	H	K	L	$I_{\varepsilon 16}$
0	8	0	10906	0	8	0	12616
0	0	6	9816	0	0	16	9265
11	0	2	9119	11	0	5	10499
10	4	0	8450	10	4	0	8435
7	0	5	7120	7	0	13	8512
0	4	0	5932	0	4	0	4768
6	7	0	3852	6	7	0	4178
2	0	0	3672	2	0	0	3244
11	0	8	3444	11	0	21	1871
3	0	5	3252	3	0	13	4813
10	12	0	3146	10	12	0	3765
18	0	3	3060	18	0	8	3130
7	0	1	3044	7	0	3	2846
8	0	3	2885	8	0	8	5761
0	0	4	2868	0	0	10	2917
10	0	0	2864	10	0	0	3247
10	3	0	2795	10	3	0	2188
5	0	2	2770	5	0	5	4572
0	0	10	2574	0	0	26	2916
4	4	0	2566	4	4	0	1988
4	0	3	2558	4	0	8	3211
6	1	0	2509	6	1	0	2176
4	0	0	2354	4	0	0	1912
2	0	3	2232	2	0	8	5000
6	0	0	2048	6	0	0	2189
0	12	0	2020	0	12	0	3501
3	0	1	2007	3	0	3	2247
1	0	2	1991	1	0	5	1948
6	6	0	1889	6	6	0	832
16	0	0	1791	16	0	0	2006
0	8	6	1697	0	8	16	3377
6	3	0	1681	6	3	0	1612
2	1	0	1644	2	1	0	674
2	6	0	1444	2	6	0	460
4	3	0	1395	4	3	0	792
10	11	0	1383	10	11	0	1762
4	1	0	1371	4	1	0	950
10	8	0	1355	10	8	0	1463
6	2	0	1297	6	2	0	766
1	0	1	1285	1	0	3	1011
5	0	8	1265	5	0	21	959
2	2	0	1236	2	2	0	463

7	0	2	1232	7	0	5	757
6	0	3	1208	6	0	8	1125
4	2	0	1195	4	2	0	529
10	5	0	1171	10	5	0	826
13	0	5	1163	13	0	13	1081
16	0	6	1155	16	0	16	880
14	0	3	1134	14	0	8	896
10	2	0	1106	10	2	0	636
6	0	10	1102	6	0	26	310
17	0	2	1102	17	0	5	586
4	5	0	1098	4	5	0	683
7	0	11	1093	7	0	29	392
10	1	0	1093	10	1	0	906
6	8	0	1085	6	8	0	1228
3	0	2	1073	3	0	5	773
15	0	2	1073	15	0	5	1055
0	2	0	1073	0	2	0	1196
8	6	0	1036	8	6	0	587
8	0	7	1008	8	0	18	544
0	8	10	987	0	8	26	1218
2	7	0	983	2	7	0	611
6	4	0	979	6	4	0	665
1	0	8	979	1	0	21	605
16	3	0	971	16	3	0	1345
20	0	0	971	20	0	0	977
14	0	6	967	14	0	16	366
10	6	0	959	10	6	0	724
16	6	0	955	16	6	0	971
0	4	10	951	0	4	26	1633
2	3	0	938	2	3	0	479
10	0	3	926	10	0	8	435
8	0	4	918	8	0	10	266
8	5	0	918	8	5	0	367
2	0	9	914	2	0	24	490
1	0	5	906	1	0	13	517
2	4	0	902	2	4	0	557
4	0	4	894	4	0	10	736
0	6	0	889	0	6	0	682
14	0	7	877	14	0	18	412
3	0	11	861	3	0	29	223
10	0	6	857	10	0	16	782
11	0	1	853	11	0	3	398
8	4	0	849	8	4	0	758
16	5	0	849	16	5	0	1140

11	0	5	845	11	0	13	379
19	0	5	840	19	0	13	468
16	0	4	836	16	0	10	498
14	0	0	832	14	0	0	963
8	0	0	828	8	0	0	405
9	0	1	812	9	0	3	703
16	7	0	808	16	7	0	718
12	4	0	796	12	4	0	499
6	0	6	792	6	0	16	756
16	8	0	779	16	8	0	1135
17	0	5	779	17	0	13	432
8	3	0	775	8	3	0	414
9	0	5	775	9	0	13	748
20	1	0	767	20	1	0	426
12	0	0	767	12	0	0	491
12	0	9	767	12	0	24	204
4	6	0	763	4	6	0	444
12	0	3	763	12	0	8	733
0	16	0	755	0	16	0	638
10	0	4	755	10	0	10	373
12	0	7	751	12	0	18	249
22	0	0	747	22	0	0	688
6	5	0	747	6	5	0	592
2	8	0	743	2	8	0	367
13	0	1	730	13	0	3	893
19	0	1	730	19	0	3	556
2	5	0	726	2	5	0	369
8	1	0	722	8	1	0	273
18	0	7	714	18	0	18	446
2	0	7	714	2	0	18	424
8	2	0	706	8	2	0	350
4	0	10	702	4	0	26	176
10	7	0	698	10	7	0	712
5	0	1	694	5	0	3	484
0	12	6	694	0	12	16	650
4	8	0	690	4	8	0	493
16	1	0	677	16	1	0	576
10	0	10	677	10	0	26	198
9	0	2	673	9	0	5	534
0	4	6	669	0	4	16	1085
4	0	7	669	4	0	18	489
9	0	4	665	9	0	11	566
7	0	8	657	7	0	21	264
6	9	0	657	6	9	0	671

5	0	5	653	5	0	13	740
4	0	6	653	4	0	16	514
0	8	4	649	0	8	10	940
12	2	0	645	12	2	0	301
6	0	4	641	6	0	10	525
17	0	1	641	17	0	3	469
6	11	0	636	6	11	0	899
0	4	4	632	0	4	10	652
6	10	0	632	6	10	0	345
14	4	0	628	14	4	0	616
18	0	0	620	18	0	0	499
4	7	0	616	4	7	0	469
15	0	4	612	15	0	11	289
11	0	4	604	11	0	11	824
10	0	7	596	10	0	18	163
12	0	4	588	12	0	10	247
1	0	6	1424	4	12	0	988
8	0	1	1248	0	3	0	934
0	0	2	1122	5	0	11	909
9	0	3	665	0	0	6	900
5	0	5	653	1	0	11	898
4	0	6	653	8	0	2	806
0	8	4	649	0	14	0	801
12	2	0	645	0	0	2	761
6	0	4	641	0	9	0	726
17	0	1	641	4	11	0	600
6	11	0	636	0	7	0	592
0	4	4	632	9	0	11	566
6	10	0	632	0	10	0	555
16	2	0	628	21	0	5	535
14	4	0	628	4	0	2	513
13	0	9	624	6	12	0	479
6	0	1	620	16	4	0	467
18	0	0	620	18	0	2	465
4	7	0	616	2	2	0	463
15	0	4	612	14	8	0	450
11	0	4	604	2	0	16	445
10	0	7	596	12	0	2	439
12	0	4	588	2	0	6	429
15	0	8	579	0	5	8	428
11	0	6	571	6	15	0	425
15	0	5	571	4	9	0	422
18	0	1	571	0	5	0	417
8	0	10	567	16	11	0	410

6	0	7	559
5	0	6	547
9	0	9	543
12	1	0	526
21	0	2	526
4	0	9	522
12	0	1	522
2	0	4	518
4	11	0	518
3	0	8	514
4	12	0	510
0	5	3	510
0	12	4	506
12	3	0	502
6	0	9	502
15	0	1	502
4	0	2	498
19	0	2	498
12	5	0	494
9	0	8	486
15	0	6	481
0	14	0	477
6	0	2	473
0	10	0	469
12	0	2	465
0	1	3	457
2	0	2	457
20	2	0	453
0	13	3	453
20	4	0	453
16	0	3	449
9	0	7	449
10	0	1	449
12	0	10	449
14	0	4	445
0	1	1	445
17	0	4	441
13	0	2	437
12	0	6	437
1	0	3	432
4	9	0	432
0	4	2	432
6	15	0	432
14	0	1	428
20	8	0	407
12	1	0	390
16	0	6	382
10	14	0	379
12	5	0	375
14	5	0	373
20	2	0	371
12	7	0	366
20	7	0	366
0	1	8	357
7	0	11	352
14	6	0	349
10	0	6	347
16	0	8	340
8	0	24	338
14	1	0	337
9	0	21	336
2	0	10	336
20	4	0	328
4	0	6	323
12	6	0	323
2	11	0	316
10	13	0	314
0	7	8	314
15	0	21	313
6	0	6	311
10	0	2	308
10	9	0	303
2	0	14	302
0	4	6	297
6	0	2	294
12	3	0	293
15	0	3	283
1	0	7	282
8	0	6	282
0	3	8	282
0	13	8	280
19	0	5	277
14	3	0	276
3	0	11	273
18	0	24	270
0	8	6	270
8	12	0	269
13	0	5	269

8	0	2	424	0	6	16	268
8	0	9	420	14	2	0	266
3	0	4	416	16	9	0	266
8	0	6	416	22	0	16	260
14	8	0	408	14	7	0	256
20	0	3	400	14	0	2	252
14	5	0	400	4	13	0	249
16	0	7	396	4	10	0	248
20	7	0	392	18	4	0	245
5	0	3	388	20	0	8	245
19	0	3	388	17	0	11	243
16	11	0	384	12	12	0	242
8	7	0	384	15	0	13	240
16	0	1	384	16	10	0	239
20	0	1	379	5	0	1	238
10	0	9	379	4	15	0	237
6	14	0	375	6	0	18	237
1	0	11	375	7	0	19	237
14	6	0	375	9	0	19	237
14	1	0	375	4	0	24	236
8	0	11	363	3	0	9	235
20	8	0	363	3	0	19	235
1	0	7	359	8	11	0	232
16	4	0	359	14	0	10	232
2	0	6	359	3	0	7	232
14	7	0	347	12	0	16	231
4	10	0	347	20	3	0	229
10	0	8	347	0	12	10	226
7	0	4	343	8	0	16	224
1	0	12	343	12	8	0	221
13	0	4	343	5	0	15	218
0	0	8	335	0	7	2	217
21	0	1	335	10	10	0	214
10	9	0	330	9	0	7	212
13	0	8	330	22	1	0	210
11	0	7	330	20	0	10	208
17	0	6	330	12	10	0	207
2	0	10	330	2	0	4	207
3	0	7	330	2	12	0	204
7	0	3	326	2	10	0	196
12	6	0	326	1	0	15	195
5	0	11	322	14	0	6	193
22	1	0	314	0	3	2	192
0	3	1	314	7	0	23	188

16	10	0	314	8	7	0	184
8	12	0	314	0	0	8	183
10	14	0	314	9	0	1	182
11	0	9	310	6	13	0	180
14	3	0	306	13	0	19	179
18	0	6	306	4	14	0	179
5	0	12	302	14	9	0	177
2	0	11	302	20	6	0	177
8	8	0	302	12	0	6	177
0	8	2	302	12	9	0	176
5	0	9	298	25	0	5	174
7	0	7	298	19	0	11	171
12	0	8	298	0	2	16	168
19	0	4	298	12	0	14	167
9	0	11	294	6	0	14	167
9	0	6	294	22	4	0	166
13	0	3	294	3	0	21	165
18	5	0	290	1	0	19	165
22	8	0	290	22	0	8	165
0	14	4	290	8	8	0	164
2	11	0	290	0	3	24	163
14	2	0	286	6	0	4	162
10	13	0	282	8	10	0	162
11	0	3	282	22	0	10	162
13	0	7	277	11	0	7	161
10	10	0	277	6	0	24	161
0	5	9	277	7	0	7	160
12	12	0	277	20	0	16	160
2	9	0	273	8	13	0	159
8	9	0	269	21	0	11	159
8	0	5	269	0	0	4	157
4	0	13	265	23	0	3	157
0	9	3	265	5	0	19	157
8	11	0	261	38	0	8	156
2	10	0	261	18	2	0	156
4	13	0	261	17	0	21	156
0	4	8	257	39	0	23	156
20	6	0	257	4	0	4	155
15	0	3	257	17	0	49	154
18	2	0	257	6	0	12	154
4	14	0	253	39	0	7	153
4	0	8	253	26	0	38	152
17	0	3	253	17	0	15	151
0	7	1	253	20	0	6	151

0	5	1	253	1	0	9	150
0	0	12	253	11	0	1	149
16	9	0	249	38	0	4	148
12	7	0	249	14	0	14	148
6	12	0	245	0	5	2	147
0	12	2	245	21	0	3	147
14	0	2	245	18	5	0	146
7	0	6	245	18	8	0	146
16	0	5	241	0	4	4	146
15	0	7	233	15	0	1	146
6	0	5	233	36	0	30	145
26	4	0	233	5	0	7	144
0	5	7	233	9	0	9	144
8	0	8	233	12	0	26	143
0	6	4	228	2	9	0	143
3	0	6	228	6	14	0	143
20	0	2	228	4	0	14	142
0	14	2	228	10	0	4	142
17	0	8	228	20	9	0	142
14	0	9	228	17	0	7	142
18	0	4	228	1	0	29	141
12	8	0	228	32	0	24	141
20	3	0	228	5	0	17	141
10	16	0	228	13	0	9	141
20	9	0	224	14	0	24	141
12	0	5	224	20	0	2	140
4	0	11	220	19	0	19	139
0	3	9	220	41	0	23	139
22	4	0	220	13	0	11	139
0	13	1	220	16	0	2	139
14	0	10	216	18	1	0	138
6	0	8	216	25	0	37	138
2	0	5	208	13	0	7	138
20	0	4	208	3	0	69	138
0	9	1	208	28	0	42	136
2	12	0	204	43	0	13	136
22	0	3	204	23	0	13	136
12	11	0	204	0	11	0	136
12	10	0	204	16	0	18	135
4	0	5	200	23	0	39	135
12	9	0	200	25	0	35	135
3	0	12	196	27	0	39	135
17	0	7	196	5	0	9	134
11	0	12	192	13	0	1	134

18	4	0	192	12	11	0	134
22	7	0	192	37	0	3	134
5	0	7	192	11	0	19	134
4	15	0	188	9	0	23	133
0	6	6	184	42	0	12	133
0	5	5	184	7	0	1	133
22	0	1	184	10	0	24	132
8	10	0	184	17	0	9	132
14	9	0	184	8	9	0	132
2	0	8	180	15	0	15	132
16	15	0	180	0	0	32	131
7	0	12	180	8	0	26	131
14	12	0	175	0	5	24	131
18	8	0	175	13	0	21	131
18	1	0	171	5	0	29	130
18	0	9	171	29	0	37	130
21	0	4	171	0	6	10	129
3	0	10	171	18	0	4	129
1	0	10	167	22	8	0	129
0	3	3	167	8	0	4	129
10	0	5	167	34	0	12	129
18	3	0	167	21	0	49	129
0	14	6	167	0	7	18	129
0	8	8	163	2	0	26	128
0	9	5	163	42	0	18	128
8	14	0	163	8	0	12	127
2	15	0	163	14	0	4	127
14	10	0	159	1	0	17	127
0	7	3	159	14	0	22	127
20	0	6	159	0	1	24	127
21	0	5	159	1	0	23	125
23	0	1	155	12	0	20	125
10	15	0	155	22	0	32	125
16	13	0	155	2	0	12	125
14	0	5	155	0	10	16	125
0	3	7	155	22	0	2	125
6	18	0	155	37	0	5	124
14	11	0	155	18	0	16	124
2	13	0	151	24	0	48	124
24	1	0	151	7	0	15	124
16	12	0	151	18	0	14	124
0	1	7	151	2	15	0	124
0	1	5	151	2	0	32	124
6	13	0	151	18	0	10	124

14	0	8	151	15	0	7	124
20	11	0	151	6	0	22	123
0	2	4	151	15	0	19	123
0	7	7	151	0	11	8	123
2	0	13	147	11	0	9	123
0	1	9	147	39	0	15	123
0	7	5	147	0	14	16	122
5	0	10	147	3	0	15	122
8	13	0	147	24	0	8	121
0	11	1	143	25	0	45	121
18	6	0	143	7	0	67	121
13	0	6	143	11	0	15	121
0	9	7	143	15	0	23	121
18	0	2	139	11	0	31	120
0	12	10	139	12	0	12	120
0	9	9	139	0	1	2	119
24	0	0	139	0	3	18	119
0	2	2	139	31	0	23	119
18	7	0	139	10	0	14	119
10	0	11	139	21	0	21	118
22	0	6	135	32	0	22	118
23	0	5	135	11	0	49	118
19	0	6	135	18	3	0	118
20	5	0	135	41	0	9	118
22	9	0	131	0	0	22	118
0	6	2	131	7	0	25	117
9	0	10	131	13	0	29	117
0	7	9	131	8	0	44	117
15	0	9	131	2	13	0	116
2	16	0	126	16	12	0	116
11	0	11	126	0	4	22	116
16	14	0	126	22	0	6	116
13	0	11	126	11	0	17	115
22	2	0	122	27	0	29	115
20	12	0	122	33	0	31	115
6	19	0	122	7	0	9	115
6	0	11	122	15	0	29	115
26	0	0	122	9	0	29	115
0	10	6	122	0	12	6	115
4	19	0	118	4	0	22	114
22	5	0	118	9	0	17	114
16	0	10	118	40	0	6	114
0	2	6	118	3	0	1	113
11	0	10	118	16	0	20	112

22	0	4	118		8	0	28	112
19	0	7	118		28	0	20	112
4	0	12	118		6	0	48	112
6	16	0	118		40	0	18	112
0	2	10	118		20	0	4	111
22	3	0	118		7	0	53	111
6	0	12	114		0	1	18	111
4	16	0	114		36	0	12	111
2	0	12	114		21	0	15	111
0	4	12	110		0	9	8	111
18	0	5	110		7	0	17	110
0	6	10	110		17	0	19	110
12	13	0	110		32	0	14	110
22	0	2	110		13	0	17	109
0	18	0	110		14	10	0	109
